# Post-remission measurable residual disease directs treatment choice and improves outcomes for patients with intermediate-risk acute myeloid leukemia in CR1

**DOI:** 10.1007/s12185-022-03441-6

**Published:** 2022-08-28

**Authors:** Lijie Han, Yilu Li, Jiaying Wu, Jie Peng, Xiaolin Han, Hongmian Zhao, Chen He, Yuanyuan Li, Weimin Wang, Mengmeng Zhang, Yafei Li, Hui Sun, Haixia Cao, Li’na Sang, Zhongxing Jiang, Jifeng Yu

**Affiliations:** 1 Department of Hematology, The First Affiliated Hospital of Zhengzhou University, Zhengzhou, China; 2 Department of Oncology, The Second Affiliated Hospital of Guizhou Medical University, Kaili, China; 3 Department of Hematology, The First Affiliated Hospital of Xinxiang Medical University, Xinxiang, China; 4 Department of Hematology, Huaihe Hospital of Henan University, Kaifeng, China; 5 Laboratory of Hematology, The First Affiliated Hospital of Zhengzhou University, Zhengzhou, China; 6 School of Foreign Languages, Henan University of Chinese Medicine, Zhengzhou, China

**Keywords:** Acute myeloid leukemia, Intermediate-risk, Measurable residual disease, Hematopoietic stem cell transplantation

## Abstract

**Objectives:**

This study retrospectively investigated in which cycle measurable residual disease (MRD) is associated with prognosis in patients in first complete remission (CR1) of intermediate-risk acute myeloid leukemia (AML).

**Methods:**

The study enrolled 235 younger patients with intermediate-risk AML. MRD was evaluated by multiparameter flow cytometry after the 1st, 2nd, and 3rd chemotherapy cycles (MRD1–3, respectively).

**Results:**

No significant association was detected after the 1st and 2nd cycles. However, the 5-year incidence of relapse was higher in the MRD3-positive group (*n* = 99) than in the negative group (*n* = 136) (48.7% vs. 13.7%, *P* = 0.005), while 5-year disease-free survival (DFS) and overall survival (OS) were lower in the MRD3-positive group than in the negative group (43.2% vs. 81.0% and 45.4% vs. 84.1%; *P* = 0.003 and 0.005, respectively). Allogeneic hematopoietic stem cell transplantation led to a lower 5-year relapse, and higher DFS and OS rates than chemotherapy in the MRD3-positive group (22.3% vs. 71.5%, 65.9% vs. 23.0%, and 67.1% vs. 23.9%; *P* < 0.001, 0.002, and 0.022, respectively), but did not affect the MRD-negative group.

**Conclusions:**

MRD3 could serve as an indicator for post-remission treatment choice and help improve outcomes for intermediate-risk AML in CR1.

**Supplementary Information:**

The online version contains supplementary material available at 10.1007/s12185-022-03441-6.

## Introduction

For adult patients with acute myeloid leukemia (AML), complete remission (CR) rates are approximately 70–80%. However, approximately 60% of these patients experience a relapse in their first CR (CR1) despite intensive chemotherapy [1]. To improve the leukemia-free survival of patients with AML, distinct therapeutic strategies for post-remission consolidation have been developed. Treatment decisions are based on cytogenetic risk profiles, and, recently, on the measurable residual disease (MRD) [2, 3].

In the treatment of intermediate-risk AML (IR-AML), post-remission relapse is a major concern; but no consensus on post-remission treatment exists. Some reports suggest that high-dose combined chemotherapy is a viable option for patients with IR-AML [4, 5]. Other studies have indicated that allogeneic hematopoietic stem cell transplantation (allo-HSCT) was associated with better survival compared with chemotherapy when applied as post-remission therapy (PRT) [6–9]. Interestingly, the HOVON/SAKKAML 42A study mentioned that MRD plays a crucial role in deciding which PRT options would be the most effective [10]. Another study, which investigated standard-risk AML in CR1 according to the second cycle MRD status, could not indicate whether allo-HSCT was significantly useful in MRD-positive cases (95% confidence interval (CI) 0.31–1.69) [11]; in the IR-AML subgroup following the GIMEMA AML1310 protocol [12], no significant differences in survival between the second cycle (one induction and one consolidation cycle) MRD-positive receiving allo-HSCT and MRD-negative receiving auto-HSCT subgroups were found. However, the selection of strategy (including allo-HSCT, auto-HSCT, and chemotherapy) for IR-AML, and the MRD cycle for deciding the therapeutic strategy remains controversial. Thus, which specific cycles of MRD are closely associated with prognosis should be clarified; this would be helpful in determining post-remission treatment strategies for IR-AML in CR1. We conducted a retrospective study examining younger adults with de novo IR-AML in CR1 who underwent either chemotherapy, auto-HSCT, or allo-HSCT as consolidation therapies in our centers.

## Methods

### Patients

Younger patients in our centers (the First Affiliated Hospital of Zhengzhou University, the First Affiliated Hospital of Xinxiang Medical University, and Huaihe Hospital of Henan University) who had a confirmed diagnosis of IR-AML between January 2015 and December 2018 were enrolled if they met the following criteria: (1) newly diagnosed with de novo IR-AML, (2) between the ages of 14 and 60 years, (3) received ≥3 cycles of chemotherapy and in CR1. The study is in accordance with the principles of the Declaration of Helsinki and was approved by the ethical committee of the First Affiliated Hospital of Zhengzhou University, the First Affiliated Hospital of Xinxiang Medical University, and Huaihe Hospital of Henan University.

### Diagnostic methods

AML was diagnosed as previously described [13]. Immunophenotyping was performed on diagnostic bone marrow (BM) aspirate samples by CD45/SSC gated 8-color flow cytometry [14]. The cytogenetic examination was performed using standard techniques. Molecular screening for fusion genes and gene mutations was performed using real-time quantitative-polymerase chain reaction, and sequence analysis was applied to all patients [15]. The classification of IR-AML was based on the National Comprehensive Cancer Net-work 2019 recommendations version 3 as follows: (1) mutated NPM1 and FLT3-ITD high; (2) wild type NPM1 without FLT3-ITD or with FLT3-ITD low (without adverse-risk genetic lesions); (3) t(9;11) (p21.3;q23.3); MLLT3-KMT2A, or (4) cytogenetic abnormalities not classified as favorable or adverse [16].

### Treatment

**Fig. 1:**
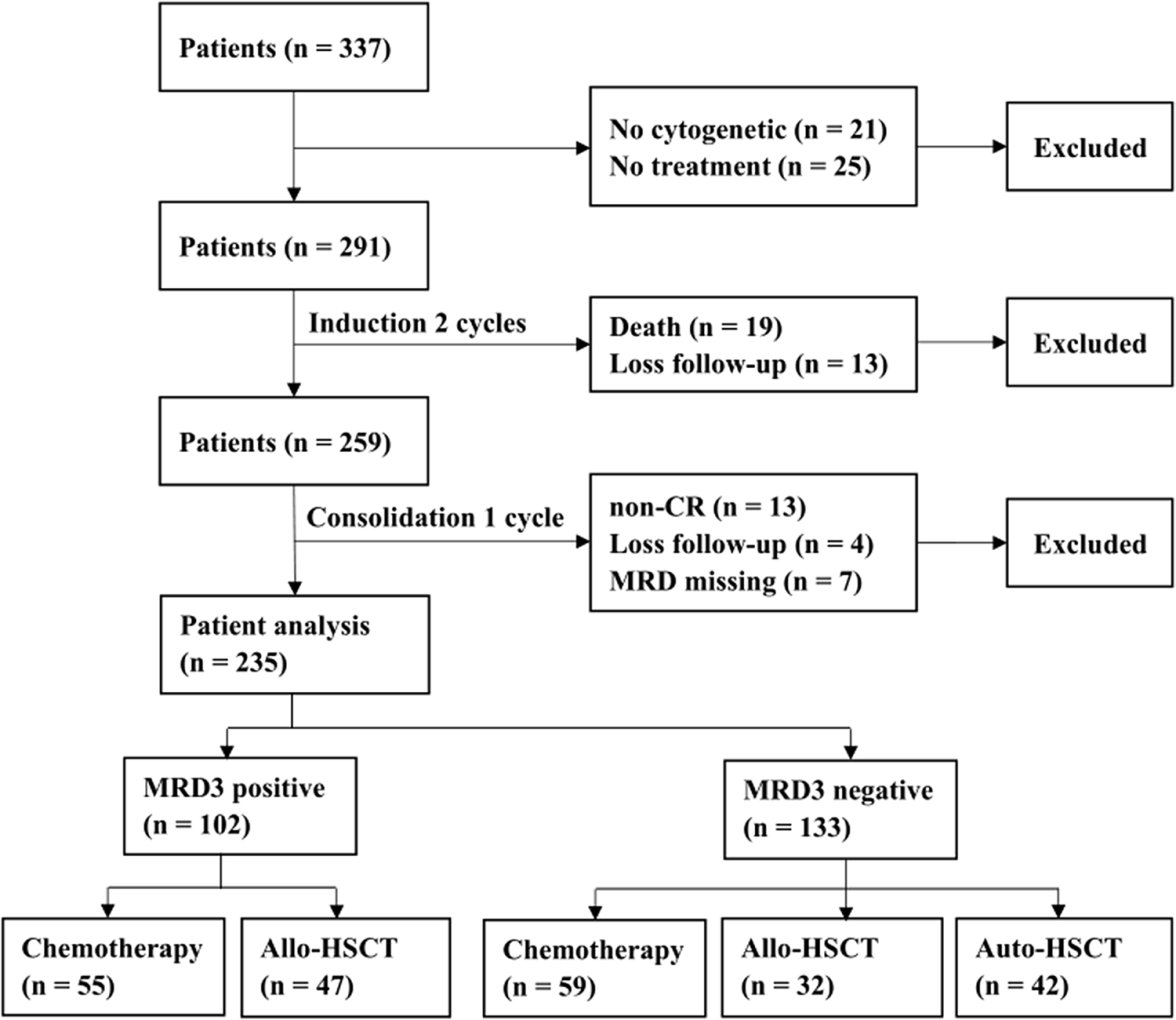
Diagram showing patients’ enrollment in this study. MRD3, measurable residual disease after the third cycle of chemotherapy (two induction cycles and one consolidation cycle)

Induction chemotherapy was administered following one of the three regimens: (1) idarubicin (8–10 mg/m^2^ for 3 days) combined with cytarabine (Ara-C, 100 mg/m^2^ for 7 days); (2) daunorubicin (45 mg/m^2^ for 3 days) in combination with Ara-C, or (3) homoharringtonine (2 mg/m^2^), Ara-C (100 mg/m^2^) and aclarubicin (20 mg) for 7 days. Induction chemotherapy was performed for two cycles if the patients achieved CR or partial remission (PR) in the first cycle. Otherwise, those who had no-remission (NR) after the first cycle received FLAG (30 mg/m^2^ fludarabine on days 1–5, 2 g/m^2^ Ara-C on days 1–5, and 300 µg G-CSF on days 0–5) or CLAG (5 mg/m^2^ cladribine on days 1–5, 2 g/m^2^ Ara-C on days 1–5, and 300 µg G-CSF on days 0–5 [17, 18]. After two cycles of induction chemotherapy, patients who were NR were administered decitabine + CAG (cytarabine, aclarubicin, and G‐CSF) or were enrolled in a clinical trial, and the CR/complete remission with incomplete count recovery (CRi) patients were given consolidation chemotherapy, which consisted of one cycle of IDAC (i.e., intermediate-dose cytarabine, 2 g/m^2^ q12h for 3 days). Subsequently, the CR/CRi patients were administered further consolidation chemotherapy (IDAC*3–4 cycles) or allo-HSCT, which consisted of matched sibling donor (MSD) transplantation, matched unrelated donor (MUD) transplantation, haploidentical donor (HID) transplantation, or auto-HSCT based on MRD and donor availability (Fig. 1). In auto-HSCT, peripheral blood stem cells were harvested after mobilization with IDAC. In allo-HSCT, Busulfan-based myeloablative conditioning regimens were administered to all patients as previously described [19].

### Treatment response definitions

CR was defined as follows: <5% BM blasts; absence of blasts with Auer rods; absence of extramedullary disease; absolute neutrophil count >1.0 × 10^9^/L; platelet (PLT) count >100 × 10^9^/L, and independence of red cell transfusions. CRi corresponded to all CR criteria except for neutropenia (<1.0 × 10^9^/L) or thrombocytopenia (<100 × 10^9^/L). PR was defined as 5% <BM blasts <25% and a decrease in the pre-treatment bone marrow blast by at least 50%. NR was defined as BM blasts ≥25%; relapse was a recurrence of ≥5% BM blasts, the reappearance of blasts in the blood, or the development of extramedullary disease, and non-CR included NR and PR as described previously [13].

### Monitoring and definition of MRD

BM samples were collected to monitor MRD using 8-color multiparameter flow cytometry (MFC) after each course of chemotherapy (induction and each cycle of consolidation). MRD was detected using a panel of eight antibody combinations [14]. LAIP (leukemia-associated immunophenotype) defined at AML diagnosis was used for MFC-MRD detection. The different-from-normal immunophenotype was performed to monitor MFC-MRD when LAIP was not available at diagnosis. The sensitivity of MFC-MRD detection was 0.1%, and any level of measurable MRD ≥0.1% was considered positive, and ≤0.1% was negative. MRD1–3 refers to the MRD after the 1st, 2nd, and 3rd cycles of chemotherapy, respectively.

### Endpoints and statistical methods

Cumulative incidence of relapse (CIR) was the primary endpoint of the study. Secondary endpoints included disease-free survival (DFS) and overall survival (OS), as estimated using the Kaplan–Meier method and compared by the log-rank test. Both CIR and DFS were measured from the date of achievement of the first remission, and OS was measured from the date of diagnosis. The χ2 analyzed categorical variables or Fisher’s exact test, and differences between distributions of continuous variables were evaluated using the Mann–Whitney *U* test. The area under the receiver operating curve constructed from logistic regression analysis was used to present the associations between MRD and relapse. Based on the cut-off value for relapse, MRD was defined as negative or positive.

The Cox proportional hazards model was applied to evaluate the association between patient characteristics and outcomes in multivariate analysis. Variables associated with relapse or survival with *P*-values less than 0.10 in univariate analysis or variables (e.g., age, high white blood cell (WBC), cycles required to achieve CR ≥2) known to influence outcomes were included in the final models. *P*-values of <0.05 whereas considered statistically significant. SPSS 22.0 (Mathsoft, Seattle, WA), GraphPad Prism 6 (GraphPad Software Inc., La Jolla, CA), and R software (http://cran.R-project.org) were used for all data analysis.

## Results

### Patient characteristics

From January 2015 to December 2018, 337 consecutive younger adult patients were diagnosed with IR-AML in our centers (Fig. 1). Of the 337 patients, 21 lacked complete cytogenetic data at diagnosis, and 25 were not treated; both were excluded from the study. Of the remaining 291 patients, 32 were excluded from this study due to death (*n* = 19) or loss to follow-up (*n* = 13) during the two cycles of induction chemotherapy. Of the remaining 259 patients, those with non-CR (*n* = 12), loss to follow-up (*n* = 4), missing MRD measures (*n* = 6), or mortality (*n* = 2) were excluded after another cycle of chemotherapy consolidation. The remaining 235 patients were enrolled.

After the first induction, 184 (78.3%) patients were MRD1-positive while 51 (21.7%) were MRD1-negative. Among the MRD1-positive patients, 91 (49.5%), 63 (34.2%), and 30 (16.3%) were administered chemotherapy, allo-HSCT, and auto-HSCT, respectively; correspondingly, 23 (45.1%), 16 (31.4%), and 12 (23.5%) MRD1-negative patients underwent chemotherapy, allo-HSCT, and auto-HSCT, respectively. After the second induction, 153 (65.1%) patients were MRD2-positive while 82 (34.9%) were MRD2-negative. Among the MRD2-positive patients, 73 (47.7%), 54 (35.3%), and 26 (17.0%) underwent chemotherapy, allo-HSCT, and auto-HSCT, respectively. Among the MRD2-negative patients, 41 (50.0%), 25 (30.5%), and 16 (19.5%) underwent chemotherapy, allo-HSCT, and auto-HSCT, respectively. After the third cycle of chemotherapy, 43.4% of patients were MRD3-positive while 56.6% were MRD3-negative. According to the MRD3 status, the patients were divided into MRD3-positive (*n* = 102) and -negative (*n* = 133) groups. The MRD3-positive group contained 55 patients in chemotherapy consolidation and 47 in Allo-HSCT subgroups; and the MRD3-negative group contained 59 in chemotherapy, 32 in allo-HSCT, and 42 in auto-HSCT subgroups (Fig. 1).

The characteristics of the patients are summarized in Table 1. The median age of the patients was 36 years, and the WBC count at diagnosis was ≥100 × 10^9^/L in 29 patients. After the first induction cycle, 181 patients achieved CR/CRi, including 51 MRD-negative cases (MRD1 <0.1%); after the second induction cycle, 215 achieved CR/CRi including 82 MRD2-negative cases; and after the third cycle, 133 (56.6%) patients were MRD3-negative. The median follow-up period for this cohort was 18 (3–72) months.

**Table 1:** Patient clinical characteristics

Characteristic	No. (%)
Age, years	
Median (range)	36 (14–60)
≤36	122 (51.9)
>36	113 (48.1)
Sex (%)	
Male	123 (52.3)
Female	112 (47.7)
WBC count at diagnosis (range) ×10^9^/L	
High WBC (>100)	29 (12.3)
WBC (≤100)	206 (87.7)
Cytogenetics and gene mutations	
Normal cytogenetics only	58 (24.7)
NPM1 and FLT3-ITD^high^	14 (6.0)
Wild NPM1 and FLT3-ITD^low^	21 (8.9)
t(8;21) with c-Kit	15 (6.4)
inv(16) with c-Kit	7 (3.0)
t(9;11)	6 (2.6)
Others without favorable and poor-risk cytogenetics and genetic lesions	114 (48.4)
Induction regimen	
IA	76 (32.3)
DA	132 (56.2)
HAA	27 (11.5)
CR/CRi achieved	
After cycle1	181 (77.0)
After cycle2	215 (91.5)
MRD- (FCM <0.1%)	
MRD1-	51 (23.7)
MRD2-	82 (34.9)
MRD3-	133 (56.6)
Consolidation	
Chemotherapy	114 (48.5)
Allo-HSCT	79 (33.6)
Auto-HSCT	42 (17.9)

*Allo-HSCT* Allogeneic hematopoietic stem cell transplantation, *Auto-HSCT* Autologous HSCT, *CR* Complete remission, *CRi* CR with incomplete blood count recovery, *MRD* Measurable residual disease, *MRD1*–*3* MRD after the 1st–3rd chemotherapy cycle, respectively

### Association between different MRD cycles and relapse and survival

**Fig. 2:**
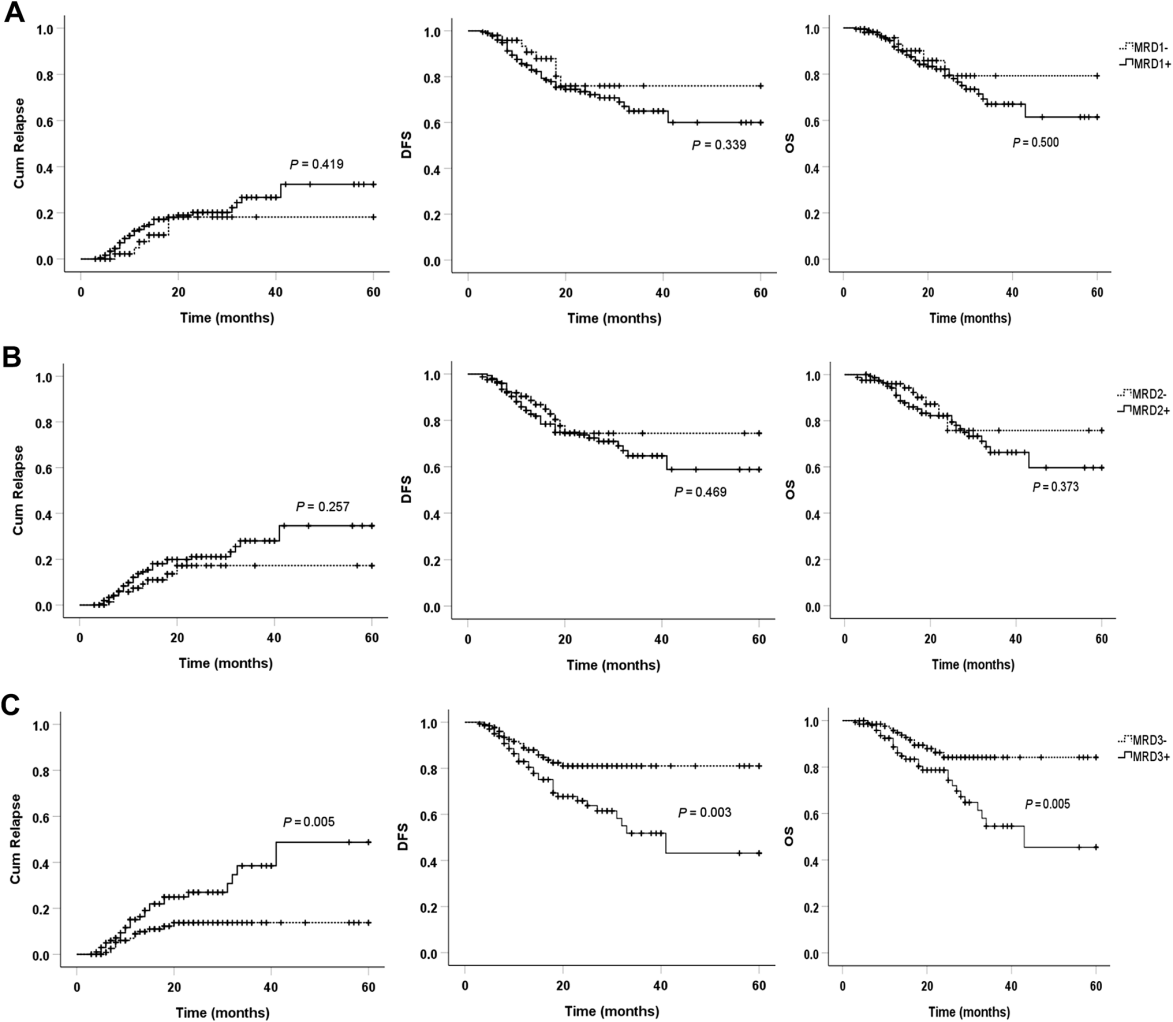
Cumulative relapse, DFS, and OS based on MRD for different chemotherapy cycles. The estimated 5-year cumulative incidence of relapse, DFS, and OS according to MRD1 **A**, MRD2 **B**, and MRD3 **C**. MRD1–3, measurable residual disease after the 1st, 2nd, and 3rd cycles of chemotherapy respectively

As shown in Fig. 2, in the context of MRD1, the estimated 5-year CIR (MRD1 positive vs. negative: 32.3% vs. 18.2%, *P* = 0.419), DFS (60.0% vs. 76.0%, *P* = 0.339), and OS (61.4% vs. 79.3%, *P* = 0.500) were not significantly different between the groups. Similarly, after the 2nd cycle (MRD2), 5-year relapse incidence, DFS, and OS were not significantly different between the groups (MRD2 positive vs. negative: 34.6% vs. 17.2%, 58.8% vs. 74.4%, and 59.7% vs. 75.8%; *P* = 0.257, 0.469, and 0.373; respectively). However, after the 3rd cycle (MRD3), the 5-year CIR was higher in the MRD3-positive group than in the negative group (48.7% vs. 13.7%, *P* = 0.005). Furthermore, the 5-year DFS and OS were lower in the MRD3-positive than in the negative group (43.2% vs. 81.0% and 45.4% vs. 84.1%; *P* = 0.003 and 0.005, respectively).

Additionally, the area under the curve (AUC) of MRD3 for predicting relapse was the largest (AUC = 0.72, *P* < 0.001, cut-off value, 0.1%), with sensitivity and specificity of 0.77 and 0.56, respectively (Figure S1). The corresponding AUCs for MRD1 and MRD2 for predicting relapse were smaller (AUC = 0.58 and 0.56, *P* = 0.111 and 0.239, respectively, Figure S1).

### Multivariate prognostic analysis

The univariate and multivariate analyses of relapse, DFS, and OS are shown in Table2. For all patients, age >36 years, MRD3 positivity, and consolidation chemotherapy (compared with allo-HSCT) was independent risk factors for relapse [*P* = 0.023, 0.001 and <0.001; hazard ratio (HR) = 2.200, 3.937 and 4.560; and 95% CI 1.116–4.338, 1.692–9.159 and 1.967–10.575, respectively]. No other factors, including high WBCs and ≥2 cycles required to achieve CR, were significantly associated with relapse in multivariate analysis (*P* > 0.05). For DFS, patient age >36 years, MRD3 positivity and consolidation chemotherapy were also independent risk factors (*P* = 0.030, 0.003 and 0.001; HR = 1.886, 2.643 and 2.872; 95% CI 1.065–3.341, 1.381–5.058 and 1.499–5.503, respectively), whereas other factors demonstrated no association with DFS (*P* > 0.05). For OS, MRD3 positivity and consolidation chemotherapy were also independent risk factors (*P* = 0.016 and 0.025; HR = 2.582 and 2.267; 95% CI 1.197–5.567 and 1.107–4.645, respectively), while patient age >36 years, high WBCs, and ≥2 cycles required to achieve CR had no significant influence (*P* > 0.05).

**Table 2:** Multivariate analysis of outcomes

Factors	Relapse		DFS		OS	
	Univariate	Multivariate (*P* (HR, 95% CI))	Univariate	Multivariate (*P* (HR, 95% CI))	Univariate	Multivariate (*P* (HR, 95% CI))
Patient age						
≤36 vs. >36 years	0.268	0.023 (2.200, 1.116–4.338)	0.348	0.030 (1.886, 1.065–3.341)	0.665	0.525 (1.235, 0.644–2.371)
High WBC vs. WBC	0.452	0.604 (1.369, 0.418–4.485)	0.634	0.847 (1.096, 0.432–2.782)	0.741	0.975 (1.017 0.358–2.884)
Cycles required to achieve CR						
1 vs. ≥2	0.709	0.523 (1.266, 0.614–2.610)	0.925	0.805 (1.082, 0.578–2.207)	0.343	0.367 (1.366, 0.693–2.693)
MRD3− vs.MRD3+	0.007	0.001 (3.937, 1.692–9.159)	0.004	0.003 (2.643, 1.381–5.058)	0.006	0.016 (2.582, 1.197–5.567)
Treatment choice						
Chem vs. Allo	0.005	<0.001 (4.560, 1.967–10.575)	0.035	0.001 (2.872, 1.499–5.503)	0.082	0.025 (2.267, 1.107–4.645)
Chem vs. Auto	0.138	0.859 (1.103, 0.372–3.237)	0.016	0.235 (1.787, 0.685–4.661)	0.056	0.400 (1.631, 0.522–5.102)
Allo vs. Auto	0.333	0.026 (0.242, 0.069–0.845)	0.792	0.371 (0.622, 0.220–1.758)	0.523	0.594 (0.719, 0.214–2.417)

*Allo* Allogeneic transplantation, *Auto* Autologous transplantation, *Chem* Chemotherapy, *CI* Confidence Interval, *DFS* Disease-free survival, *HR* Hazard ratio, *MRD* Measurable residual disease, *MRD3* MRD after the third chemotherapy cycle, *OS* Overall survival

### Interaction between MRD and beneficial effects of HSCT in CR1

In this study, 42 patients who were MRD3 negative underwent auto-HSCT in CR1. Among the other patients, consolidation chemotherapy and allo-HSCT were administered to 114 and 79 cases respectively. The allo-HSCT subgroup included 34 patients (43.0%) with a human leukocyte antigen (HLA)-MSD transplant (10/10 HLA-matched), 21 patients (26.6%) with a matched or mismatched unrelated donor transplant (10/10–9/10 HLA-matched), and 24 patients (30.3%) with an HLA-mismatched related donor transplant (1/10–5/10 HLA-mismatched).

**Fig. 3:**
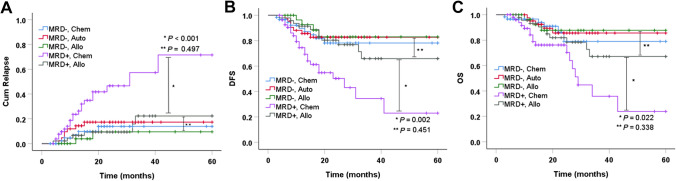
Interaction between MRD3 and beneficial effects of HSCT in AML with CR1. The estimated cumulative incidence of relapse **A**, DFS **B**, and OS **C** according to the MRD level after the 3rd cycle of chemotherapy (≥0.1% or <0.1%) in patients with intermediate-risk acute myeloid leukemia in the first complete remission. *Allo* allogeneic hematopoietic stem cell transplantation, *Auto* autologous hematopoietic stem cell transplantation, *Chem* Chemotherapy consolidation, *DFS* Disease-free survival, *MRD* Measurable residual disease, *OS* Overall survival

The interaction between MRD1-3 and HSCT and its effects on patient outcomes were assessed. Among chemotherapy, allo-HSCT, and auto-HSCT subgroups of MRD1-positive and -negative patients, the estimated CIR, and survival are shown in the Supplementary Material; the 5-year cumulative OS was not significantly different (*P* > 0.05, Figure S2). Among chemotherapy, allo-HSCT, and auto-HSCT subgroups of MRD2-positive and -negative patients, CIR and survival are shown in the Supplementary Material; the estimated 5-year OS was not significantly different (*P* > 0.05, Figure S2). The effects of MRD3 and HSCT on patient outcome were also evaluated (Fig. 3). The results indicated that outcomes were improved by allo-HSCT (*n* = 47) compared with consolidation chemotherapy (*n* = 55) for patients with MRD3 positivity. For MRD3-negative patients, no significant difference between different therapies was found in 5-year cumulative relapse (13.9% vs. 9.5% vs. 17.3% in chemotherapy, allo-HSCT, and auto-HSCT subgroups, respectively; *P* = 0.469, Fig. 3). Similarly, there was no statistically significant difference in the estimated 5-year DFS and OS rates among the three subgroups (DFS: 78.1% vs. 83.0% vs. 82.7%; OS: 79.0% vs. 87.7% vs. 85.6%; *P* = 0.805 and 0.940; respectively, Fig. 3). However, for MRD3-positive patients, the 5-year CIR was lower in the allo-HSCT than in the chemotherapy subgroup (22.3% vs. 71.5%, *P* < 0.001, Fig. 3); the 5-year cumulative DFS and OS were higher in the allo-HSCT than in the chemotherapy subgroup (65.9% vs. 23.0%, and 67.1% vs. 23.9%; *P* = 0.002 and 0.022, respectively, Fig. 3).

## Discussion

AML is a heterogeneous clonal disease with dismal survival, largely due to high relapse incidence [20]. Although more than 70% of patients will achieve CR1 after induction chemotherapy, many may relapse [21]. In previous studies, MRD status was proven to be an independent prognostic predictor for oncologic outcomes [10, 22, 23]. The precise timepoint at which MRD is associated with prognosis remains controversial. In this study, we demonstrated that MRD3-positive patients (i.e., after the third chemotherapy cycle, two inductions and one consolidation) were more likely to relapse compared to those with MRD3 negativity in the population of younger patients with de novo IR-AML. Additionally, MRD3-positive patients had lower 5-year DFS and OS. However, these unfavorable outcomes were ameliorated more effectively by allo-HSCT than chemotherapy. Therefore, our results indicated that MRD3 (after the third cycle of chemotherapy) was an appropriate indicator of PRT choice for IR-AML in CR1.

For patients with IR-AML who have achieved CR after induction therapy, additional PRT is an essential component for maintaining a disease-free status. Three PRT alternative strategies, including conventional chemotherapy, auto-HSCT, and allo-HSCT, could be chosen as consolidation therapy for post-induction. The centers have different choices that depend on factors such as patient characteristics, availability of a donor, and doctors’ discretion [24–27]. Auto-HSCT has higher availability and a lower rate of non-relapse mortality than allo-HSCT. However, it is associated with a higher relapse rate, due to a lack of a graft-versus-leukemia effect [28]. Allo-HSCT following a myeloablative conditioning regimen has been considered an effective option for reducing the risk of relapse for patients with AML in CR1, particularly in younger patients with MRD-positive status. These patients should preferentially be offered myeloablative rather than reduced-intensity conditioning allo-HSCT [29–31]. Nevertheless, the timepoint at which MRD is most closely associated with prognosis remains controversial. A few studies showed that MRD after cycle 1 or 2 was associated with prognosis, while another study suggested that MRD was an independent prognostic factor after several cycles by next-generation sequencing (such as pre-transplantation) [10, 11, 32].

Several factors influence MRD status and the subsequent outcomes of patients with IR-AML, including the chemotherapy intensity, infection, HSCT application, and survival [33–35]. Recently, a study indicates that high-intensity relative to low-intensity chemotherapy results in higher rates of MRD-negative cases [35]; another study indicates that induction therapy of IDAC with daunorubicin and omacetaxine mepesuccinate increases survival in patients with AML aged 15–55 years relative to conventional-dose cytarabine [36]. Luca Maurillo reported that MRD negativity is not less in the standard-dose group as compared to high-dose cytarabine after both induction and consolidation [37]. Three doses of cytarabine consolidation (1.5 g/m2, 2 g/m^2^, and high-dose cytarabine (HiDAC) 3 g/m^2^) were administered to intermediate- and poor-risk AML patients, and showed no significant differences in DFS and OS; however; septic shock was significantly higher after HiDAC 3 g/m^2^ administration as compared to the IDAC regimen [38]. The present study indicated that MRD direction and HSCT selection in the PRT were vital for improving the survival of IR-AML patients.

In our study, we explored the role of MRD1-3 in directing the selection of PRT for patients with de novo IR-AML in CR1, although the selection for transplant was dependent on MRD status and donor availability. As demonstrated by our results for patients who were MRD3 negative, outcomes did not differ among high-dose chemotherapy, auto-HSCT, and allo-HSCT treatment. Venditti et al. [12] also reported that auto-HSCT exerts an effect similar to that of allo-HSCT on patients with IR-AML and MRD negativity. However, for our patients with MRD3 positivity, allo-HSCT yielded a better outcome than consolidation chemotherapy; it significantly decreased relapse rates and increased DFS and OS. Additionally, different outcomes were not found among the MSD, MUD, and HID transplant subgroups, although the sample sizes were limited. This agrees with results from recent studies, which have also indicated that clinical outcomes for HID are equivalent to MSD and MUD transplantation [39–42]. Therefore, consolidation chemotherapy and auto-HSCT could be considered a valuable alternative after induction therapy, which may be preferred for patients with IR-AML and MRD3 negativity in the absence of a suitably matched donor. In the presence of low resources, allo-HSCT should be reserved for patients with IR-AML and MRD3 positivity.

Our results also demonstrated that MRD3, but not MRD1 or MRD2 was closely related to relapse and survival. MRD3 could be interpreted as insufficient ‘depth of remission’ from the first two chemotherapy cycles, and patients should undergo or prepare for allo-HSCT to prevent relapse and protect organs at the time of the fourth chemotherapy cycle. We also found that a 0.1% cut-off value for MRD was appropriate to discriminate between patients with significantly different CIRs. In addition, a recent study by Paiva et al. [43] indicated that a cut-off value of 0.1% was suitable to discriminate patients with different relapse rates, although other reports showed different values [44, 45].

This study had some limitations. First, our study was not randomized, and the number of patients was not large. Second, the possibility of treatment bias exists, as patients with MRD positivity may be less likely to receive auto-HSCT due to the high rate of relapse [46]. In addition, older patients were not included in this study because of their inability to receive HSCT. Therefore, larger, prospective randomized clinical trials are required to validate, modify, or disprove our results with more certainty.

In summary, our results indicate that MRD3, rather than MRD1 or MRD2, could be used, not only as a potent predictor of outcomes, but also as an indicator of optimal subsequent treatment strategies for patients with de novo IR-AML in CR1. The findings of this study also suggest that patients with IR-AML could be further categorized based on MRD3 status, which could improve personalized PRT.

## Supplementary Information

Below is the link to the electronic supplementary material.

Supplementary file1 (DOCX 305 KB)
