# Supplementary material for: Post-remission measurable residual disease directs treatment choice and improves outcomes for patients with intermediate-risk acute myeloid leukemia in CR1
Source: Int J Hematol. 2022 Aug 28;116(6):892–901. doi: 10.1007/s12185-022-03441-6 (PMC9668963; doi:10.1007/s12185-022-03441-6)
Supplement: Supplementary file 1 — Supplementary file1 (DOCX 305 KB) [file 12185_2022_3441_MOESM1_ESM.docx]

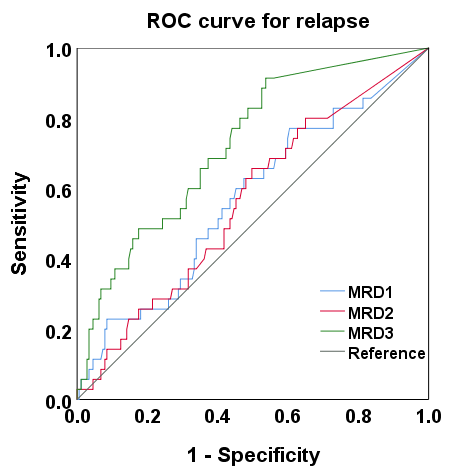


**Figure S1. MRD levels of different chemotherapy cycles predicted relapse of acute myeloid leukemia.** The area under the receiver operating curve [(ROC), AUC] of MRD3 for predicting relapse was the largest (AUC = 0.72, *P* < 0.001, cutoff value, 0.1%), with sensitivity and specificity of 0.77 and 0.56, respectively. While, the AUCs of MRD1 and MRD2 for predicting relapse were smaller (AUC = 0.58 and 0.56, *P* = 0.111 and 0.239, respectively). MRD, measurable residual disease; MRD1~3, MRD after the 1^st^~3^rd^ chemotherapy cycle, respectively.


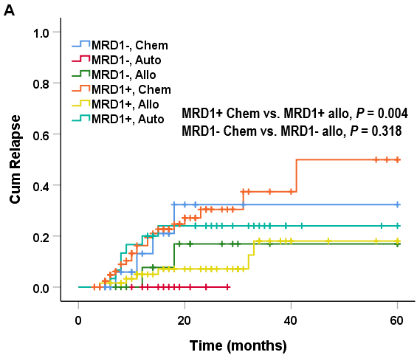

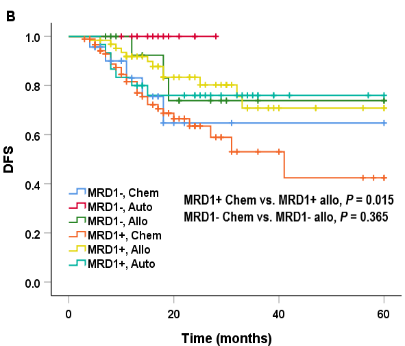

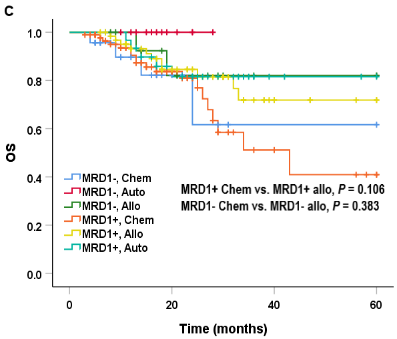


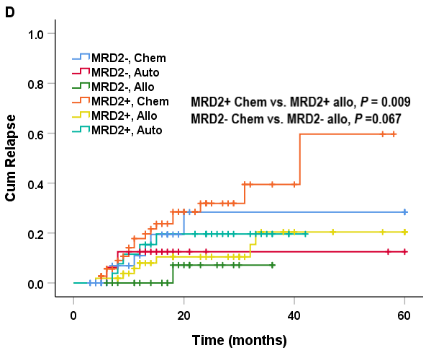

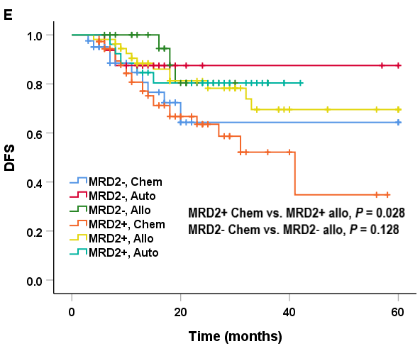

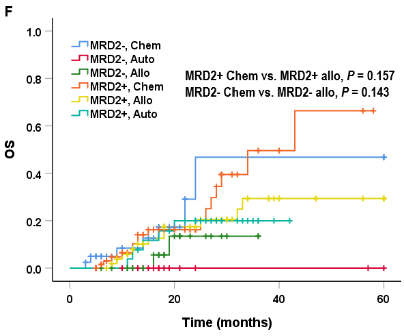


**Figure S2. Interaction between MRD1-2 and beneficial effects of HSCT in AML with CR1.** The **estimated** cumulative incidence of relapse (A, D), DFS (B, E), and OS (C, F) according to the MRD1 and MRD2 level after the 1^st^ and 2^nd^ cycle of chemotherapy (≥ 0.1%, or < 0.1%) in patients with intermediate-risk acute myeloid leukemia in the first complete remission. In MRD1 positive group (After the first induction), the 5-year CIR was lower in the allo-HSCT than in the chemotherapy subgroup (18.0% vs. 49.9%; P = 0.004, A), and the **estimated** 5-year cumulative DFS was higher in the allo-HSCT than in the chemotherapy subgroup (70.8% vs. 42.4%, P = 0.015, B). But the 5-year OS was not identified difference between allo-HSCT and chemotherapy groups (40.9% vs. 71.9%, P = 0.106, C). In MRD1-negative group, there was also no statistically significant difference in the 5-year CIR, DFS and OS rates among the three subgroups (CIR: 32.3% vs. 16.9% vs. 0.0%; DFS: 64.8% vs. 73.8% vs. 100.0%; OS: 61.7% vs. 82.1% vs. 100.0%; P = 0.155, 0.127 and 0.211; respectively, A-C). In MRD2-positive group (After the second induction), the 5-year CIR was lower in the allo-HSCT than in the chemotherapy subgroup (20.4% vs. 59.6%; P = 0.009, D), and the 5-year cumulative DFS was higher in the allo-HSCT than in the chemotherapy subgroup (69.5% vs. 34.8%, P = 0.028, E). But the 5-year OS was not identified difference among the groups (33.6% vs.70.7% vs. 80.1%, P = 0.313, F). In MRD2 negative group, there was also no significant difference in the 5-year CIR, DFS and OS rates among the three subgroups (CIR: 28.4% vs.7.1% vs. 12.5%; DFS: 64.3% vs. 80.4% vs. 87.5%; OS: 53.2% vs. 86.6% vs. 100.0%; P = 0.205, 0.256 and 0.083; respectively, D-F). Allo, allogeneic hematopoietic stem cell transplantation; Auto, autologous hematopoietic stem cell transplantation; Chem, Chemotherapy consolidation; DFS, disease-free survival; MRD, measurable residual disease; OS, overall survival.
